# Antibacterial and Antibiofilm Efficacies of Cell-Free Supernatant of *Dubosiella newyorkensis* Against *Pseudomonas fluorescens* and Its Application in Food Systems

**DOI:** 10.3390/foods15030581

**Published:** 2026-02-05

**Authors:** Ailin Wang, Meihan Zhang, Yunqi Gu, Yuanhang Cheng, Ningbo Qin, Xiaodong Xia

**Affiliations:** 1 State Key Laboratory of Marine Food Processing and Safety Control, National Engineering Research Center of Seafood, School of Food Science and Technology, Dalian Polytechnic University, No. 1 Qinggongyuan, Ganjingzi District, Dalian 116034, China; wal0630@163.com (A.W.); 18845570473@163.com (M.Z.); 13470398760@163.com (Y.G.); Neilcheng1012@gmail.com (Y.C.); 2 Dalian Xinghai Bay Laboratory, Dalian 116034, China; 3 Department of Food Science and Nutrition, The Hong Kong Polytechnic University, Hong Kong 999077, China

**Keywords:** *Dubosiella newyorkensis*, *Pseudomonas fluorescens*, cell-free supernatant, biofilm formation, gene expression

## Abstract

*Pseudomonas fluorescens* is a primary spoilage bacterium in aquatic products. Due to its strong ability to adhere to surfaces and form persistent biofilm, it poses a persistent challenge to food safety. Therefore, developing strategies to effectively inhibit biofilm formation holds significant research value. *Dubosiella newyorkensis*, a recently identified probiotic, has gained growing attention for its distinctive physiological features and potential functional benefits. Although various probiotic-derived cell-free supernatants (CFSs) have been explored for food preservation, the application of *D. newyorkensis* CFS against aquatic spoilage bacteria, and particularly its specific mechanism against *P. fluorescens* biofilm, has not been previously reported. Increasing evidence indicates that CFS from probiotic can influence microbial behavior, including biofilm development. In this study, we investigated the ability of *D. newyorkensis* CFS to inhibit *P. fluorescens* biofilm formation. The CFS treatment impaired bacterial growth and motility, lowered surface hydrophobicity, reduced self aggregation, and consequently hindered biofilm formation. Furthermore, CFS markedly decreased bacterial adhesion to food and contact surfaces. RT-qPCR analysis revealed that key genes associated with biofilm regulation were also significantly suppressed. Taken together, these results demonstrate that *D. newyorkensis* CFS exerts both antibacterial and antibiofilm effects against *P. fluorescens*. These findings provide a sound basis for exploring its application as a natural biopreservative to enhance the microbial safety and extend the shelf life of aquatic food products.

## 1. Introduction

Microbial spoilage remains a major obstacle facing the aquatic food industry, not only leading to a decline in product quality but also causing significant economic losses [1]. Among the primary specific spoilage organisms (SSOs) found in chilled foods, *Pseudomonas fluorescens* stands out for its exceptional adaptability to environmental fluctuations [2]. The spoilage capacity of *P. fluorescens* arises from a combination of biochemical reactions, notably lipolysis, proteolysis, and lecithinase synthesis. These metabolic pathways are driven by the secretion of heat-stable extracellular enzymes, such as alkaline metalloproteases and lipases, which remain active even under refrigeration conditions [3,4]. Through lipase-mediated hydrolysis, lipid molecules are rapidly broken down into unsaturated glycerol and fatty acids, compounds that impart undesirable sensory traits, including rancid or bitter notes [5]. In parallel, proteolytic enzymes degrade muscle proteins into peptides and free amino acids, which further decompose into biogenic amines and volatile organic compounds [6]. This biochemical cascade contributes to off-flavors, such as bitterness and ammoniacal odors, and induces textural deterioration by causing tissue softening and the loss of functional properties. Equally noteworthy is the cooperative interaction between *P. fluorescens* and other spoilage-associated microbes, which significantly exacerbates the overall spoilage process. For instance, co-cultivation with Hibiscus-derived bacteria was shown to elevate viable cell counts by 0.59 log CFU/g relative to monoculture conditions [7]. Parallel to this microbial synergy, the concentrations of total volatile basic nitrogen (TVB-N) and putrescine rose by 22.7% and 31.4%, respectively, hastening the deterioration of shrimp broth. Overall, these observations indicate that the spoilage activity of *P*. *fluorescens* exhibits multifaceted characteristics. There is an urgent need for precise and effective control measures to mitigate its negative impact on food ecosystems.

*P*. *fluorescens* significantly accelerates aquatic food spoilage by forming robust biofilm. Its mechanisms include quorum sensing regulation, extracellular matrix barriers, and enhanced enzyme activity [8]. Knockout experiments demonstrated that *P*. *fluorescens* regulated biofilm formation and motility through iron carrier-dependent mechanisms. The absence of iron carriers significantly reduces its putrefactive potential [9]. In a study on refrigerated *Siniperca chuatsi*, the research reported that cyclic di-GMP (c-di-GMP) signaling promotes biofilm development by activating polysaccharide synthesis genes (*psl* and *alg*) via diguanylate cyclase (DGC) regulation, while concurrently repressing flagellar gene expression [10]. These changes led to significant increases in total volatile basic nitrogen (TVB-N), thiobarbituric acid reactive substances (TBARS), and carbon disulfide accumulation in the fish filets. Furthermore, the physical barrier of the biofilm enhanced the bacterium’s environmental resistance [11]. *P. fluorescens* poses a significant threat to food safety due to its remarkable adaptability and complex regulatory mechanisms,, particularly during cold-chain transportation and packaging. While strain-specific variability exists, the core physiological traits that enable *P. fluorescens* to dominate in cold-chain environments, such as psychrotolerance, biofilm robustness, and spoilage enzyme production, are highly conserved. Consequently, the findings derived from this specific strain provide a representative model for understanding spoilage dynamics. Furthermore, the fundamental interactions observed here are likely to be extrapolatable across various aquatic food matrices, as the physicochemical properties of these environments consistently favor the persistence and biofilm formation of this versatile pathogen.

*Dubosiella newyorkensis* is a Gram-negative bacterium. Its unique biological characteristics and metabolic diversity have attracted significant attention. Studies have shown that *D. newyorkensis* contributed to the regulation of host intestinal homeostasis by producing a range of bioactive metabolites, including short-chain fatty acids (SCFAs) and amino acid derivatives. Notably, *D. newyorkensis* synthesizes significant amounts of propionic acid and L-lysine, which play pivotal roles in maintaining the balance between regulatory T cells (Treg) and T helper 17 (Th17) cells, thereby enhancing intestinal barrier integrity and immune modulation [12]. These metabolites not only promote microbial stability in the gut but also improve the host’s resilience against dietary toxins and pathogenic insults. Moreover, *D. newyorkensis* has been shown to enhance the immune tolerance of dendritic cells (DCs) via activation of the aryl hydrocarbon receptor (AhR)-indoleamine 2,3-dioxygenase 1 (IDO1)-kynurenine (Kyn) signaling pathway [13]. Beyond these host-immune functions, the metabolites produced by *D. newyorkensis*, particularly SCFAs and organic acids like propionic acid, possess well-documented broad-spectrum antimicrobial properties [12]. In non-host environments, such as food matrices, these compounds can effectively penetrate bacterial membranes and disrupt cellular functions, thereby inhibiting quorum sensing and biofilm formation [14]. This suggests that the metabolic arsenal of *D. newyorkensis* has direct application potential as a natural biopreservative, extending its utility beyond gut health to food safety.

Aquatic food is widely valued for its natural origin, nutritional richness, and multifunctional properties. Through deep processing, aquatic food products can achieve enhanced flavor, improved safety, and greater consumer appeal [15]. However, due to their high protein content and moisture levels, aquatic food products are highly susceptible to colonization by SSOs, such as *Pseudomonas* and *Shewanella*, leading to reduced shelf life and considerable economic losses. Under refrigerated storage conditions, controlling these spoilage microorganisms remains a major challenge in maintaining the quality of aquatic foods. Consequently, increasing attention is being directed toward developing effective strategies to prevent contamination by *P. fluorescens*. With the continuous growth in global demand for aquatic foods, the development of preservation technologies that combine high-efficiency anti-corrosion properties has become a critical bottleneck in the industry’s advancement. *D. newyorkensis* has demonstrated diverse bioactivities and produced diverse functional substances. It may be applicable in food products and has the potential to become a good probiotic in the future. However, the mechanisms by which the cell-free supernatant (CFS) of *D. newyorkensis* interacts with *P. fluorescens* remain poorly understood. To our knowledge, this is the first study to investigate the inhibitory effects of *D. newyorkensis* cell-free supernatant against *P. fluorescens* and elucidate the underlying mechanism, providing a theoretical foundation for its application in aquatic food preservation.

## 2. Materials and Methods

### 2.1. Bacterial Strain and Culture

The strain of *P. fluorescens* ATCC 13525 was obtained from the China Industrial Microbial Storage Centre. *D. newyorkensis* ATCC TSD-64 was purchased from Tsto Biotech Co., Ltd. (Ningbo, Zhejiang, China). *P. fluorescens* was incubated in LB at 35 °C and *D. newyorkensis* was cultured anaerobically in Modified Reinforced Clostridial Broth at 37 °C for 24 h.

### 2.2. Preparation CFS from D. newyorkensis

*D. newyorkensis* was incubated in an anaerobic incubator (10% H_2_, 10% CO_2_, 80% N_2_) for 24 h to ensure a live bacteria concentration of 1 × 10^9^ CFU/mL. After reaching the optimal growth conditions, the culture medium of *D. newyorkensis* was centrifuged (12,000 rmp/min, 10 min, 4 °C), and the precipitate of the organisms was discarded, and passed through a 0.22 μm sterile membrane to obtain CFS [16]. To preserve its natural antimicrobial properties, the CFS was used without pH adjustment. All batches were prepared under strictly standardized culture conditions to ensure compositional reproducibility, yielding the CFS of pH of 6.62 ± 0.02, protein content of 614.95 ± 14.39 ng/mL, and organic acid concentration of 8.53 ± 0.23 mmol/L. The supernatant was stored at −80 °C before use.

### 2.3. Growth Curve

*P. fluorescens* cultures were transferred into 96-well microplates supplemented with CFS at gradient concentrations of 0%, 25%, 50%, 75%, and 100% (*v*/*v*). Unless indicated otherwise, subsequent analyses were carried out under these five experimental conditions. The cultures were maintained at 35 °C for 48 h, and optical density at 600 nm was automatically recorded every two hours using a growth monitoring system (Bioscreen C, Oy, Helsinki, Finland) [17]. The incubation temperature was set at 35 °C, as this corresponds to the growth temperature for *P. fluorescens*, simulating the worst-case scenario for microbial spoilage in food systems [18].

### 2.4. Motility Activity

Swimming and swarming tests were performed with semi-solid LB plates containing 0.3% agar and BHI plates containing 1.5% agar, respectively [19]. 2 μL of overnight cultured *P. fluorescens* (10^9^ CFU/mL) were inoculated onto 0.3% LB plates and 1.5% BHI plates, respectively, and incubated at 35 °C for 24 h. The diameters of bacterial motility zones were measured with a chemical imaging system.

### 2.5. Biofilm Formation

Biofilm formation was analyzed by crystalline violet staining. CFS was added to *P. fluorescens* suspension (10^8^ CFU/mL), and 200 μL was inoculated into 96-well plate [20]. The culture solution was removed, rinsed gently with PBS, dried for about 20 min, and stained with 200 μL of 0.1% (*w*/*v*) crystal violet, and the reaction was carried out for 20 min and rinsed three times with PBS. The stained biofilm was air-dried for 30 min and resuspended in 200 μL of 33% (*v*/*v*) glacial acetic acid for 20 min, and the absorbance at 570 nm was read by an enzyme meter.

### 2.6. Field Emission Scanning Electron Microscopy (FE-SEM)

A 500 μL suspension of *P. fluorescens* (10^9^ CFU/mL) was mixed with an equal volume of CFS and inoculated into a 24-well plate lined with cell crawls on the bottom and incubated for 24 h [21]. The samples were fixed with 2.5% glutaraldehyde at 4 °C for 4–6 h, followed by sequential 15 min dehydration steps in ethanol (30, 50, 70, 80, 90, and 100%). After overnight air-drying, biofilm morphology was visualized using FE-SEM (JSM-7800 F, JEOL, Tokyo, Japan).

### 2.7. Metabolic Activity

The metabolic activity of *P. fluorescens* biofilm was assessed using the CCK-8 assay [22]. Biofilm grown in 96-well plates was rinsed with PBS and incubated with 10% CCK-8 solution at 35 °C for 1 h. Absorbance at 450 nm was measured with a microplate reader to quantify metabolic activity. Optical density at 630 nm was measured prior to CCK-8 addition to normalize for biofilm biomass.

### 2.8. Live–Dead Cells Staining

The staining working solution was prepared according to the instructions, and the bacterial suspension was mixed with the CFS and transferred to an incubator at 35 °C for 4 h of incubation [23]. After the reaction was completed, the fluorescence of the samples was observed. The proportions of live (green) and dead (red) cells were quantified by measuring fluorescence intensities using ImageJ 1.42 software (National Institutes of Health, Bethesda, MD, USA).

### 2.9. Extracellular Polymeric Substances (EPS) Assay

*P. fluorescens* biofilm was grown on sterile glass coverslips in 24-well plates for 24 h at 35 °C [24]. The biofilms were then rinsed with PBS, resuspended in 0.01 M KCl, and subjected to ice-bath sonication to release EPS. After centrifugation at 5000× *g* (4 °C, 5 min), the supernatant was filtered (0.22 μm) to obtain the crude EPS. Extracellular polysaccharides and proteins in the EPS fraction were quantified using the phenol-sulfuric acid (490 nm) and Bradford (562 nm) assays, respectively.

For microscopy, biofilm was dual-stained with DAPI (10 mg/L) for nucleic acids and FITC-ConA (50 μg/mL) for polysaccharides. After washing, the samples were imaged by fluorescence microscopy, with DAPI and FITC-ConA signals appearing blue and green, respectively.

### 2.10. Gene Expression Analysis

Total RNA was isolated from *P. fluorescens* cells exposed to the CFS using the SteadyPure Universal RNA Extraction Kit, following the manufacturer’s instructions. The purity and concentration of the extracted RNA were verified and standardized using nanospectrophotometer (ND-ONE, Thermo Fisher, Waltham, MA, USA) [25]. cDNA was synthesized via reverse transcription with the PrimeScript™ RT Master Mix Kit I (Takara, Japan). RT-qPCR amplification was subsequently carried out using the TB Green^®^ Premix Ex Taq™ II Kit (Takara, Japan). The threshold cycle (Ct) values were automatically recorded, and the relative expression levels of target genes were determined using the 2^−ΔΔCt^ method, with *16SrRNA* serving as the internal reference gene. The sequences of the primers used for amplification are listed in Table 1.

### 2.11. Determination of Self-Aggregating and Surface Hydrophobicity

A suspension of *P. fluorescens* (1 × 10^7^ CFU/mL) was treated with varying proportions of CFS and incubated at 35 °C for 24 h. Following incubation, self-aggregation was assessed by measuring the OD_600_ of the supernatant and the vortexed suspension [26]. For surface hydrophobicity, cells were harvested by centrifugation (12,000× *g*, 10 min), washed with PBS, and the initial OD_600_ was measured. The bacterial suspension was then mixed with xylene in a 2:1 ratio (*v*/*v*), vortexed for 60 s, and incubated for 30 min to allow phase separation. The OD_600_ of the aqueous phase was measured, and surface hydrophobicity was calculated.

### 2.12. Biofilm Formation on Content of Different Foods and Surfaces

Biofilm formation was evaluated on multiple substrates: stainless steel coupons (5 × 2 × 0.1 cm), food-grade glass slides (5 × 2 × 0.1 cm), lettuce pieces (2 × 2 × 0.1 cm), and fish cubes (tilapia: 2 × 2 × 2 cm). All materials were immersed in LB broth supplemented with varying concentrations of CFS and inoculated with 1 mL of *P. fluorescens* suspension (10^7^ CFU/mL). After 24 h of incubation at 35 °C, samples were rinsed twice with PBS to remove non-adherent cells. Biofilm was then detached by vortexing each sample for 2 min in PBS containing sterile glass beads. The resulting suspensions were serially diluted and plated to quantify viable biofilm-associated cells [27].

### 2.13. Statistical Analysis

All experiments were conducted in triplicate. Statistical analyses were performed using GraphPad Prism version 8.0.2. Data are expressed as the mean ± SD. Group means were compared by one-way ANOVA. A *p*-value of less than 0.05 was considered statistically significant.

## 3. Results

### 3.1. Antimicrobial Activity of CFS In Vitro

**Figure 1:**
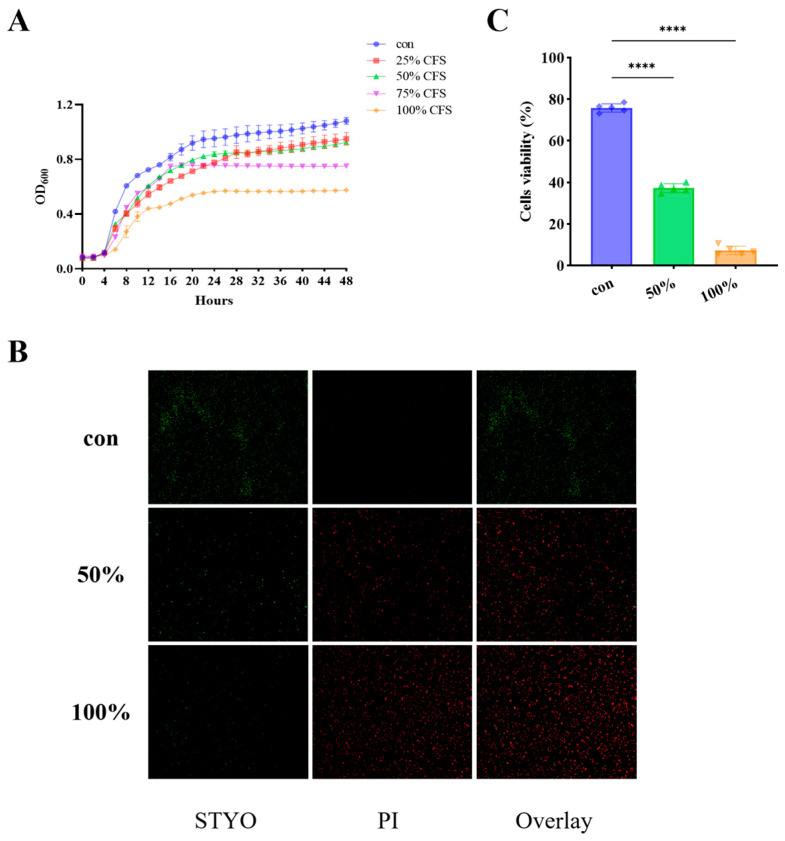
CFS effectively inhibited the growth of *P. fluorescens* and impacted its viability. Growth curves (**A**), live and dead cell staining (**B**,**C**). Colors and different shapes represent different groups and sample sizes. **** *p* < 0.0001.

The antimicrobial potential of the CFS derived from *D. newyorkensis* against *P. fluorescens* was evaluated, and the results are presented in Figure 1. Co-incubation of *P. fluorescens* with varying concentrations of CFS markedly suppressed bacterial growth in a concentration-dependent manner. After 24 h of incubation, the OD_600_ of untreated cultures reached 0.95 ± 0.14, whereas exposure to 100% CFS resulted in a significantly lower OD_600_ value of 0.57 ± 0.01, indicating a strong inhibitory effect. Fluorescence microscopy using live/dead staining (Figure 1B) further confirmed this observation: cells in the control group predominantly emitted green fluorescence, reflecting intact membranes, while CFS-treated cells displayed a pronounced increase in red fluorescence, suggesting extensive membrane disruption. The intensity of red fluorescence increased proportionally with CFS concentration, demonstrating that the bactericidal activity of the supernatant intensified in a dose-dependent manner.

### 3.2. CFS Reduced P. fluorescens Motility Ability

**Figure 2:**
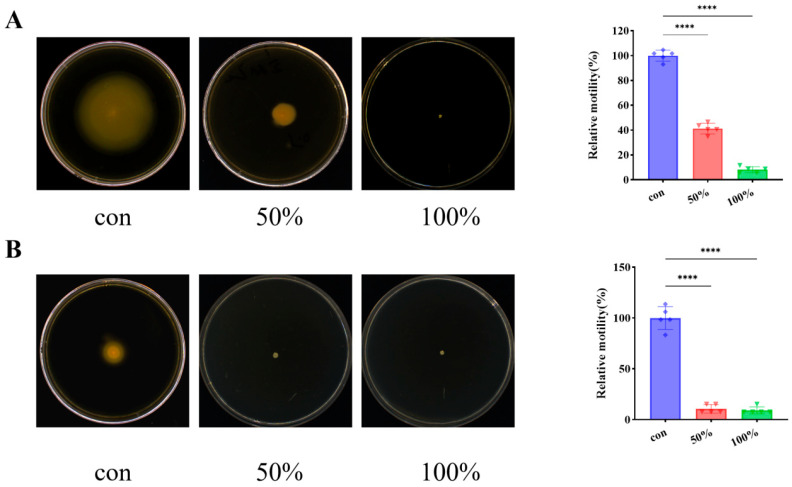
CFS inhibited *P. fluorescens* motility. (**A**) Swimming; (**B**) Swarming. **** *p* < 0.0001.

Figure 2 demonstrates the effect of *D. newyorkensis* CFS on the motility of *P. fluorescens*. In Figure 2A, treatment with 50% CFS reduced swimming to 41.27% of the control, while 100% CFS reduced it to 8.14%. This represents a significant, concentration-dependent inhibition of swimming (*p* < 0.05). Similarly, as seen in Figure 2B, the swarming diameter was reduced to 10.61% of the control with 50% CFS and to 9.09% with 100% CFS, indicating a significant suppression of swarming (*p* < 0.05). At the molecular level, this marked inhibition suggests that the CFS may interfere with flagellar synthesis or chemotaxis regulation [28]. By impairing these motility mechanisms, the CFS effectively hinders the initial surface exploration and reversible attachment stages of biofilm development, thereby preventing the transition to irreversible adhesion and subsequent mature biofilm formation.

### 3.3. CFS Reduced Cell Surface Hydrophobicity and Self-Aggregation

**Figure 3:**
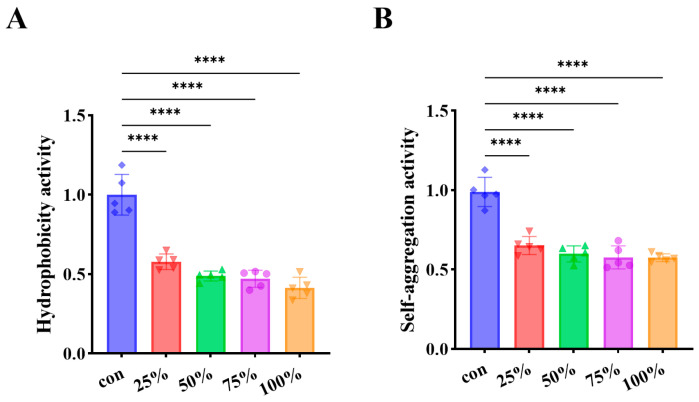
Inhibition of *P. fluorescens* surface hydrophobicity and self-aggregation by CFS. (**A**) Surface hydrophobicity; (**B**) Self-aggregation. **** *p* < 0.0001.

Cell surface hydrophobicity and self-aggregation are key physicochemical properties that facilitate bacterial adhesion and subsequent biofilm establishment. As illustrated in Figure 3A, treatment with the CFS markedly diminished the surface hydrophobicity of *P. fluorescens*, and the degree of inhibition exhibited a clear dose-dependent trend. These findings indicate that the CFS derived from *D. newyorkensis* effectively interferes with the hydrophobic interactions essential for bacterial attachment. Furthermore, as shown in Figure 3B, the auto-aggregation ability of *P. fluorescens* was substantially reduced following CFS exposure, with inhibition intensifying as CFS concentration increased. Additionally, alterations in cell surface charge (zeta potential) may also contribute to impaired adhesion by increasing electrostatic repulsion between cells and surfaces. Together with reduced hydrophobicity, this would further weaken the initial attachment [29]. Together, these results suggest that the reduction in both surface hydrophobicity and self-aggregation contributes to the overall suppression of biofilm formation by *P. fluorescens*.

### 3.4. CFS Suppressed Biofilm Development and Reduced Metabolic Activity

**Figure 4:**
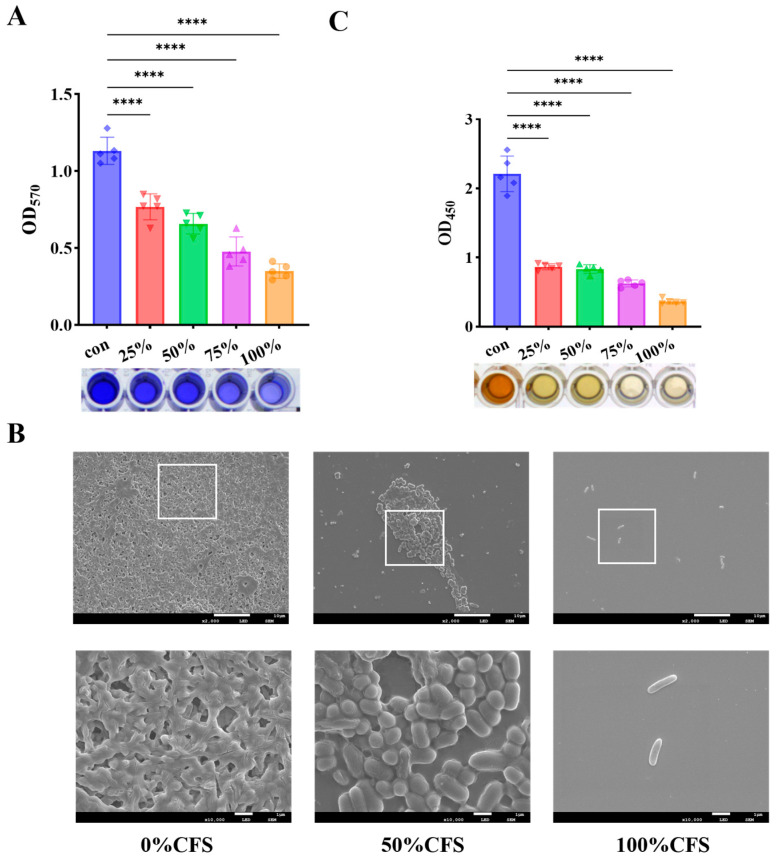
CFS reduced biofilm formation and metabolic viability in *P. fluorescens*. (**A**) Biofilm formation; (**B**) SEM; (**C**) Metabolic viability. **** *p* < 0.0001.

The protocol involved co-incubating the CFS with bacterial inoculations to assess its capacity for biofilm inhibition (Figure 4). As shown in Figure 4A, the inhibitory effect of CFS on *P. fluorescens* biofilm formation increased with CFS concentrations, exhibiting a clear dose-dependent trend. Scanning electron microscopy revealed that the control samples displayed compact and multilayered biofilm structures, whereas those treated with CFS exhibited sparse cell clusters. Furthermore, CFS treatment substantially diminished the metabolic activity of the biofilm cells, resulting in an 83.49% reduction at the highest CFS concentration (100%). These findings indicate that *D. newyorkensis* CFS effectively interferes with the initial establishment and development of biofilm, likely by impairing cellular metabolism and preventing the formation of a structured biofilm matrix.

### 3.5. CFS Decreased the Secretion of EPS

**Figure 5:**
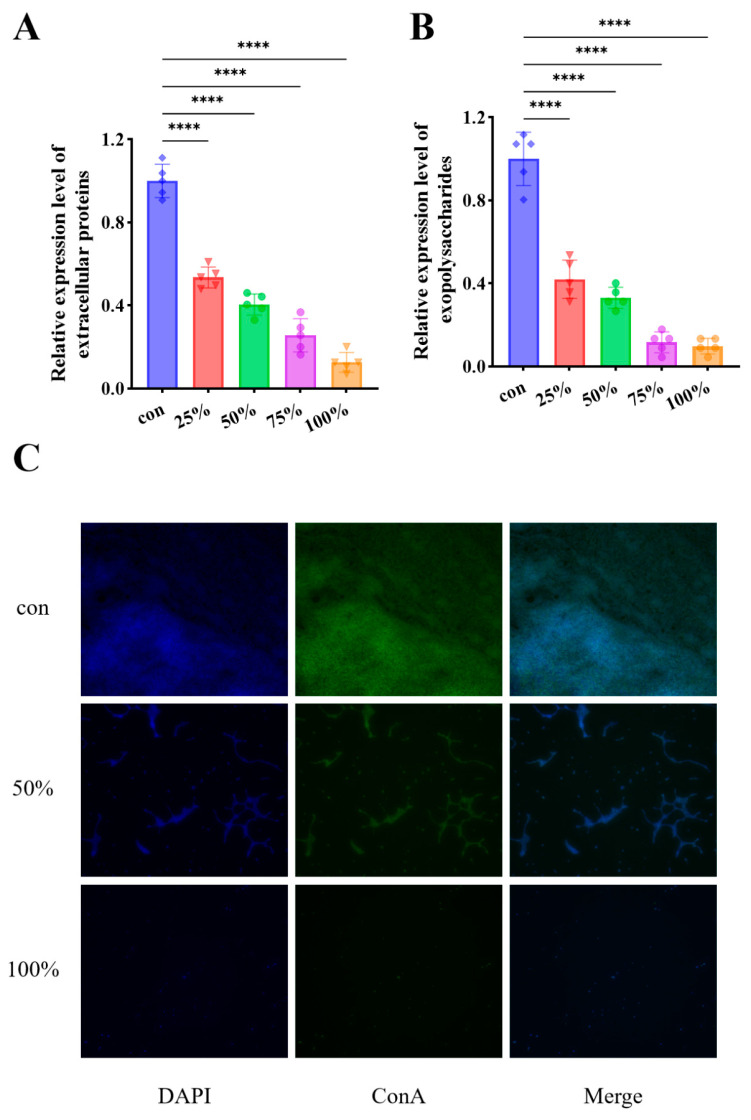
CFS decreased extracellular polymer levels in *P. fluorescens*. (**A**) Exopolysaccharides; (**B**) Extracellular proteins and (**C**) Fluorescence microscopy images. **** *p* < 0.0001.

To investigate the influence of the CFS on the synthesis of biofilm extracellular matrix components, the levels of extracellular polysaccharides and proteins within the EPS of *P. fluorescens* biofilm was quantified. Exposure to CFS led to a pronounced reduction in both constituents compared with the untreated control. As illustrated in Figure 5A, the concentration of extracellular polysaccharides decreased markedly (*p* < 0.001) in a dose-dependent pattern, showing a reduction of approximately 46.50–87.42% after 24 h of incubation. Likewise, CFS treatment significantly diminished the extracellular protein content of *P. fluorescens* (*p* < 0.001), with decreases ranging from 58.04% to 90.18% relative to the control group (Figure 5B). Fluorescence microscopy further revealed a concurrent decline in both biofilm mass and polysaccharide accumulation, where bacterial cells appeared blue and polysaccharide components were visualized in green (Figure 5C). The reduction in EPS appears to be both a direct consequence of the bioactive metabolites interfering with EPS synthesis pathways and a secondary effect resulting from the suppression of bacterial growth and metabolic activity [30]. By disrupting the structural integrity of the EPS matrix and reducing its production, the CFS effectively compromises the stability and protective function of the *P. fluorescens* biofilm. Beyond the simple inhibition of biosynthesis, the marked reduction in EPS components may also be attributed to alternative mechanisms. For instance, the CFS might possess or induce enzymatic activities that actively degrade pre-existing polysaccharides and proteins within the matrix, leading to the disintegration of the biofilm architecture. Additionally, the suppression of EPS could be a stress-mediated response, where sub-lethal environmental stress caused by the CFS triggers bacterial cells to downregulate energy-intensive EPS production or alters the expression of genes associated with matrix stability [24]. These potential degradation or stress-response pathways could act synergistically with inhibited synthesis to compromise biofilm integrity. Collectively, these results demonstrate that CFS from *D. newyorkensis* impairs the formation of the extracellular matrix, thereby weakening the structural stability of *P. fluorescens* biofilm.

### 3.6. CFS Modulated the Expression of Biofilm and Motility Associated Genes in P. fluorescens

**Figure 6:**
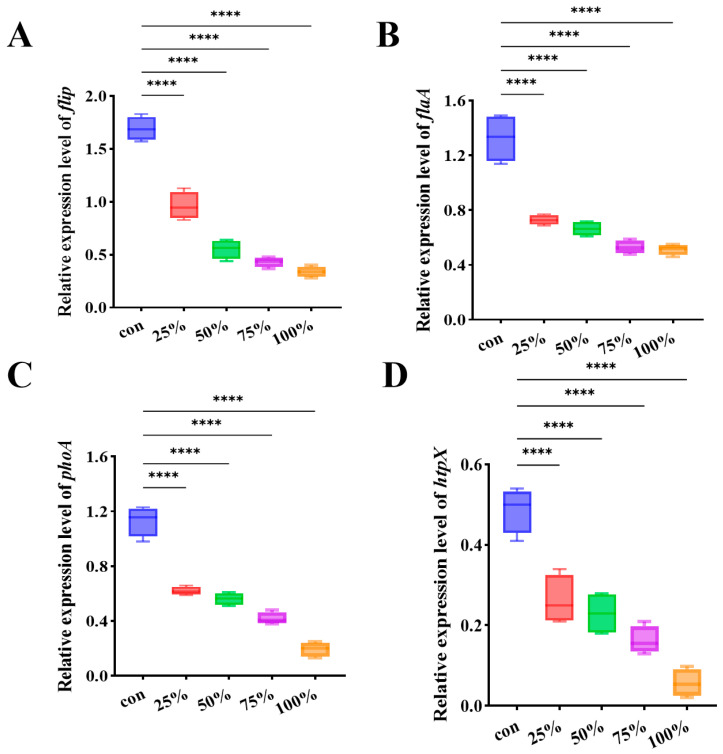
CFS down-regulated genes associated with biofilm formation in *P. fluorescens*. (**A**) *flip*; (**B**) *flaA*; (**C**) *phoA*; (**D**) *htpX*. **** *p* < 0.0001.

To elucidate the molecular mechanisms underlying the inhibitory effect of the CFS, the transcriptional responses of four genes associated with biofilm development and motility, *flip*, *flaA*, *phoA*, and *htpX*, were examined in *P. fluorescens*. As illustrated in Figure 6, exposure to CFS markedly suppressed the expression of all four target genes compared with the untreated control. These results suggest that CFS interferes with the regulatory network controlling surface attachment, flagellar synthesis, and stress response, thereby weakening the bacterium’s capacity for motility and biofilm formation. More specifically, the concurrent downregulation of these genes may indicate a disruption in key signaling pathways that govern the biofilm life cycle. For instance, the suppression of *flaA* and *flip*, which are often modulated by quorum sensing (QS) systems, suggests that the CFS could interfere with QS-mediated cell-to-cell communication, thereby preventing the population-wide behavioral changes required for biofilm maturation [31]. Furthermore, the reduced expression of biofilm-associated genes points to the potential involvement of the second messenger c-di-GMP, a central regulator of the transition between motile planktonic and sessile biofilm states [32]. However, it is important to acknowledge that while these transcriptional changes provide valuable insights into the regulatory mechanisms affected by CFS, they do not directly confirm corresponding changes in protein expression or enzymatic activity.

### 3.7. CFS Inhibited Biofilm Development of P. fluorescens on Diverse Food and Material Surfaces

**Figure 7:**
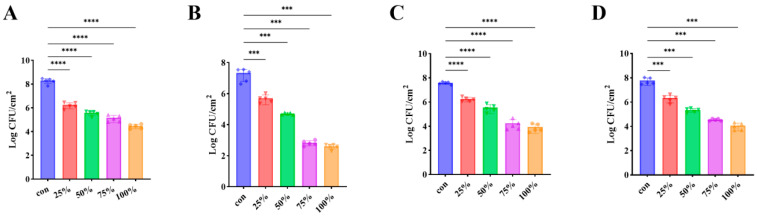
CFS reduced *P. fluorescens* biofilm formation on different contact surfaces and food products. (**A**) Lettuce; (**B**) Fish; (**C**) Stainless steel (SS) coupons; (**D**) Glass. *** *p* < 0.001 and **** *p* < 0.0001.

To assess the inhibitory potential of the cell-free supernatant (CFS) against surface-associated biofilm, *P. fluorescens* was allowed to form biofilm on several substrates commonly encountered in food processing environments, including lettuce, fish, stainless steel, and glass. As presented in Figure 7, the application of CFS markedly suppressed biofilm accumulation across all tested surfaces. Quantitatively, the viable biofilm cell counts on lettuce and fish decreased from 8.25 log CFU/cm^2^ and 7.19 log CFU/cm^2^ in the control group to 4.44 log CFU/cm^2^ and 2.58 log CFU/cm^2^, respectively, following CFS treatment. Similarly, biofilm densities on stainless steel and glass were reduced from 7.57 log CFU/cm^2^ and 7.71 log CFU/cm^2^ to 3.65 log CFU/cm^2^ and 3.98 log CFU/cm^2^, respectively. It is worth noting that these substrates vary significantly in physical properties, such as surface roughness, wettability, and nutrient microenvironments, factors that typically influence bacterial adhesion and the susceptibility of biofilms to antimicrobial agents. Despite these heterogeneous surface characteristics, which can lead to variations in biofilm structural integrity and protection, the CFS exhibited robust inhibitory efficacy across all materials. These results demonstrate that CFS exerts a pronounced antibiofilm effect across heterogeneous substrates, highlighting its potential for controlling biofilm contamination in both food matrices and contact surfaces.

## 4. Discussion

In recent years, biofilm formed by *P. fluorescens* in the food industry have become a persistent source of contamination, forming a three-dimensional structure by adhering to the interfaces of stainless steel, plastics, and aquatic tissues [33], which not only secretes lipases and proteases to accelerate food spoilage, but also serves as a breeding ground for cross-contamination of pathogens. Traditional disinfectants (such as chlorine-based agents and quaternary ammonium compounds) have limited efficacy in prevention and control. This is because they struggle to penetrate the EPS barrier of biofilm and readily induce the emergence of drug-resistant strains [34]. Therefore, it is urgent to develop new control strategies. Natural inhibitors (curcumin derivatives, essential oils such as carvacrol) and synthetic analogs can effectively block the las/rhl signaling system of *P. fluorescens*, inhibit biofilm formation, and are less likely to induce resistance [35]. Among the physical interventions, reactive oxygen radicals generated by low-temperature plasma (CP) can efficiently kill *P. fluorescens* and penetrate the biofilm, while specific wavelengths of blue light/UV-LED can achieve residue-free disinfection by non-thermal effect, which is suitable for continuous treatment in production lines [36]. In addition, although phage therapy and bacteriocins have host-specific advantages [37], their stability in complex food matrices, large-scale application and regulatory barriers still need to be broken through. When compared to GRAS preservatives and single-target bacteriocins, the CFS evaluated in this study presents a promising alternative. While traditional chemical preservatives often require high concentrations that may adversely affect sensory qualities, and bacteriocins like nisin exhibit narrow-spectrum activity that may be ineffective against Gram-negative spoilage bacteria without chelators, the multi-component nature of *D. newyorkensis* CFS appears to offer a synergistic inhibitory effect [38,39]. This complex composition likely contributes to a robust anti-biofilm efficacy at potentially lower active concentrations, enhancing its suitability for integration into food preservation strategies.

It is worth noting that the specific cultivation conditions play a crucial role in determining the bioactivity of the CFS. Since metabolite composition can vary significantly under different oxygen environments, the potent antibiofilm effects observed here are strictly linked to our anaerobic culture settings. Therefore, to ensure batch-to-batch consistency and efficacy, it is imperative to strictly adhere to these standardized anaerobic parameters during production. We report CFS that disrupt biofilm formed by *P. fluorescens* in food and on surfaces. And it inhibits the biofilm formation process by affecting bacterial motility to modulate bacterial surface interactions and preventing bacteria from attaching to surfaces. The present findings offer novel strategies for developing biofilm disruptors and propose a targeted control technology against the dominant spoilage bacteria in aquatic products. This approach enables precise intervention against these bacteria, effectively delaying the spoilage process. Thereby, it enhances storage stability and improves overall product quality and safety.

Motility is a critical determinant in the initial adhesion phase of biofilm development. Directed motility enhances microbial migration toward potential attachment surfaces and facilitates the irreversible adhesion process by promoting transient cell–substrate and cell–cell interactions [40]. In recent years, increasing attention has been given to the use of natural compounds to impair bacterial motility as a strategy to inhibit biofilm formation. For example, plant-derived phenolic compounds, including vanillin and butyric acid, have been shown to inhibit *Staphylococcus aureus* biofilm formation significantly. This inhibition is achieved by reducing motility and suppressing EPS production [41]. In addition to plant extracts, other natural agents such as antimicrobial peptides have also demonstrated antibiofilm activity by targeting bacterial motility [42]. Consistent with these findings, the present study showed that the CFS of *D. newyorkensis* effectively inhibited the motility of *P. fluorescens*, thereby contributing to reduced biofilm formation. Furthermore, the reduction in motility may have broader implications for attenuating the spoilage phenotype. Beyond adhesion, flagellar motility is closely linked to the secretion of extracellular hydrolytic enzymes, such as proteases and lipases, which are primary drivers of nutrient acquisition and sensory degradation in aquatic foods [43,44]. Impaired motility could restrict the ability of *P. fluorescens* to efficiently colonize nutrient-rich microenvironments or disperse enzymes effectively throughout the food matrix. Therefore, the CFS-induced suppression of motility may not only hinder biofilm establishment but also indirectly mitigate the overall spoilage potential of this organism.

Biofilm formation in *P. fluorescens* is a complex, multistage process involving initial bacterial attachment, cell proliferation, extracellular matrix secretion, and biofilm maturation [11]. This process is tightly regulated by various genes and signaling pathways. Additionally, plant-derived compounds like allicin, an active component of garlic extract, have been shown to impair biofilm development by interfering with genes involved in extracellular polysaccharide synthesis [45,46]. Flavonoids such as baicalin exert antibiofilm effects by modulating the activity of the global stress response regulator RpoS, leading to downregulation of biofilm-associated genes [47].

Numerous virulence factors contribute to the pathogenicity of *P. fluorescens*. In this study, CFS treatment significantly downregulated the expression of genes, including *flip*, *flaA*, *phoA*, and *htpX*. These genes are critically involved in biofilm formation and environmental adaptation. Specifically, the simultaneous downregulation of flagellum-related genes (*flip* and *flaA*) impairs bacterial motility and initial surface adhesion [48]. Inhibition of *phoA*, a phosphate stress-responsive gene, may interfere with the modification of phosphoryl groups in EPS, thereby destabilizing the biofilm matrix [9]. Notably, *htpX*, which encodes a membrane-localized protease essential for repairing stress-induced damage to membrane proteins, is also suppressed. Its down-regulation may render the bacteria more susceptible to membrane-targeted disinfection strategies [49]. These findings are in line with previous reports. For example, carvacrol disrupts the spatial conformation of *FlaA*-encoded flagellar filament proteins through hydrophobic interactions, resulting in defective flagellar assembly [50]. Gallic acid directly inhibits *HtpX* protease activity, thereby blocking the heat-shock response pathway and enhancing the efficacy of disinfectants against mature biofilm [51].

## 5. Conclusions

This study provides the first demonstrating that the CFS derived from *D*. *newyorkensis* exerts a pronounced inhibitory influence on the formation of *P*. *fluorescens* biofilm. Treatment with the CFS not only curtailed the overall biomass of the biofilm but also disturbed its structural integrity, leading to a marked reduction in the metabolic viability. Furthermore, the CFS notably diminished the production of EPS, thereby weakening the structural stability of the biofilm matrix. In summary, these findings demonstrate the promise of *D. newyorkensis* CFS as a natural and sustainable agent to control *P. fluorescens* biofilm formation on food-contact surfaces.

However, this study has several limitations that should be acknowledged. The specific identity of the active metabolites responsible for the observed antibiofilm effects remains to be elucidated. Additionally, the efficacy of the CFS was evaluated under in vitro conditions, and storage trials using actual food systems have not yet been conducted. Future work should therefore aim to isolate, identify, and characterize the key bioactive compounds employing advanced analytical approaches such as metabolomic or proteomic profiling, as well as bioactivity-guided fractionation. Furthermore, studies must elucidate their precise mechanisms of action and evaluate their performance in real-world food preservation scenarios. These efforts will be critical for translating this discovery into practical applications for food preservation and industrial biofilm management.

## Figures and Tables

**Table 1:** Primers for RT-qPCR.

Genes	Primer	Sequence (5’-3’)
*16SrRNA*	Forward	ACCGTCAAGGGACAAGCA
	Reverse	GGGAGGCAGCAGTAGGGA
*htpX*	Forward	TTCGGCTTCAACGGGTTCATGG
	Reverse	GGTGCTGGTGCTCATCTTCGC
*phoA*	Forward	TGTATAACCGCTCGCCGTTTATCG
	Reverse	GTAGAAGTGCCCGTGCTGGATTG
*flaA*	Forward	CTGGTATGAGTCGCCTTAG
	Reverse	CATTTGCGGTGTTTGGTTTG
*flip*	Forward	AACGGCATTCCAGATCGGCTTC
	Reverse	AATCAGCGGCGACAGCATCATC

## Data Availability

The main data supporting the findings are included in the article. Data are available for research purposes on reasonable request.
